# A randomised phase 1 study to investigate safety, pharmacokinetics and impact on gut microbiota following single and multiple oral doses in healthy male subjects of SMT19969, a novel agent for *Clostridium difficile* infections

**DOI:** 10.1186/s12879-015-0759-5

**Published:** 2015-02-25

**Authors:** Richard Vickers, Neil Robinson, Emma Best, Roger Echols, Glenn Tillotson, Mark Wilcox

**Affiliations:** Summit PLC, 85b Park Drive, Milton Park, Abingdon, Oxford, OX14 4RY UK; Microbiology, Leeds Teaching Hospitals & University of Leeds, Old Medical School, Leeds General Infirmary, Leeds, LS1 3EX UK; 753 Westport Road, Easton, CT 06612 USA; TranScrip Partners LLC, Downingtown, PA USA

**Keywords:** SMT19969, Phase 1, Safety, Gut microbiota, CDI, CDAD

## Abstract

**Background:**

*Clostridium difficile* infection (CDI) is a leading cause of diarrhoea in health care settings with symptoms ranging from mild and self-limiting to life threatening. SMT19969 is a novel, non-absorbable antibiotic currently under development for the treatment of CDI. Here we report the results from a Phase I study.

**Methods:**

A double-blind, randomized, placebo-controlled study assessing safety and tolerability of single and multiple oral doses of SMT19969 in healthy volunteers. Pharmacokinetic assessments included blood and faecal sampling. The effect of food on systemic exposure and analysis of the gut microbiota were also included.

**Results:**

Fifty-six healthy male subjects were enrolled. Following single oral doses of up to 2,000 mg in the fasted state, plasma concentrations of SMT19969 were generally below the lower limit of quantification. In the fed state levels ranged from 0.102 to 0.296 ng/mL after single dosing and after repeat dosing at Day 10 from 0.105 to 0.305 ng/mL. Following single and multiple oral doses of SMT19969, mean daily faecal concentrations increased with increasing dose level and were significantly above the typical MIC range for *C. difficile* (0.06-0.5 μg/mL). At 200 mg BID, mean (± SD) faecal concentrations of 1,466 (±547) μg/g and 1,364 (±446) μg/g were determined on days 5 and 10 of dosing respectively. No notable metabolites were detected in faeces. Overall, all doses of SMT19969 were well tolerated both as single oral doses or BID oral doses for 10 days. The majority (88%) of adverse events (AEs) were classified as gastrointestinal disorders and were mild in severity, resolving without treatment. The gut microbiota was analysed in the multiple dose groups with minimal changes observed in the bacterial groups analysed except for total clostridia which were reduced to below the limit of detection by day 4 of dosing.

**Conclusions:**

Oral administration of SMT19969 was considered safe and well tolerated and was associated with negligible plasma concentrations after single and multiple doses. In addition, minimal disruption of normal gut microbiota was noted, confirming the highly selective spectrum of the compound. These results support the further clinical development of SMT19969 as an oral therapy for CDI.

**Trial registration:**

Current Controlled Trials. ISRCTN10858225.

## Background

*Clostridium difficile* infection (CDI) is a leading cause of diarrhoea particularly in hospitals and other health care settings. The disease is primarily associated with prior antibiotic use causing a disruption to normal gut microbiota allowing *C. difficile* to proliferate [1], with symptoms ranging from mild self-limiting diarrhoea to more serious manifestations including pseudomembranous colitis, toxic megacolon, bowel perforation, sepsis, and death [2]. Recent years have seen a rise in the severity and incidence of CDI [3], which is partly due to the emergence of hyper-virulent strains of the bacteria such as the BI/NAP1/027 strain [4]. A particular concern is recurrence of infection which occurs in approximately 25% of patients [5], with each recurrent episode associated with increased risk of further recurrence and disease severity [6].

Until recently treatment options for CDI were mainly limited to oral metronidazole and vancomycin, neither of which are optimal particularly in the treatment of recurrent infections [7]. Both agents can cause further disruption to the gut microbiota during therapy, which may promote recurrent episodes of CDI [8,9]. Vancomycin achieves faecal concentrations sufficient to inhibit even Gram-negative organisms, such as *Bacteroides fragilis* [10]. Metronidazole is highly absorbed from the gastrointestinal tract and has a spectrum of activity encompassing normal anaerobic bowel microbiota [11,12]. In addition, *C. difficile* isolates with reduced susceptibility to metronidazole have been reported [13], and both agents are associated with acquisition or overgrowth of organisms such as vancomycin-resistant enterococci (VRE) [14]. Fidaxomicin, a minimally absorbed macrocyclic antibiotic with a narrower spectrum of activity, has recently been approved in the United States and Europe, and extends the treatment options for CDI [15,16]. However, additional antibiotics are still needed that reduce rates of recurrent disease, particularly those associated with infection due to hyper-virulent strains, and to preserve use of vancomycin for the treatment of serious systemic Gram-positive infections.

SMT19969 (2, 2′ bis(4-pyridyl) 3H, 3′H 5,5′ bibenzimidazole) is a novel antibiotic currently under development for the treatment of CDI. *In vitro* studies have reported *C. difficile* MIC_90_ values of 0.125 μg/mL and 0.25 μg/mL [17,18]. SMT19969 displays targeted activity against *C. difficile* with little or no activity against both Gram-negative and most Gram-positive aerobes and anaerobes [17]. Preclinical animal studies have reported negligible systemic exposure and a favourable safety profile [19]. In the hamster model of CDI, oral administration of SMT19969 has been shown to be superior to vancomycin, conferring significant protection from initial infection and recurrent disease [20].

The objective of the study described here was to assess the safety, tolerability and pharmacokinetics of SMT19969 in healthy volunteers following single and multiple oral doses. In addition, faecal samples were analysed for changes in gut microbiota composition following repeat oral administration.

## Methods

### Study drugs

The Active Pharmaceutical Ingredient (API) was manufactured according to Good Manufacturing Practices and supplied by Cambridge Major Laboratories (Weert, Netherlands) along with batch numbers, TSE statements and Certificates of Analysis. Covance (Leeds, UK) supplied the appropriate grade of diluent (water) and the placebo (magnesium hydroxide carbonate). Investigational Medicinal Product (IMP) was prepared in bottles as a 30 mL suspension containing the appropriate weight of SMT19969 or magnesium hydroxide carbonate and stored at room temperature. In order to maintain the blinded status of the study the placebo suspension was identical in appearance to the SMT19969 suspension.

### Design and objectives

The study was conducted in a Clinical Research Unit (CRU) by Covance CRU Ltd (Leeds, UK) who also performed the PK analysis and reporting. Clinical laboratory evaluations were performed by Covance Clinical Pathology Services (Harrogate, UK). Gut microbiome analysis was performed at Microbiology Department, Leeds Teaching Hospitals & University of Leeds (Leeds, UK).

With the exception of Group A, which was conducted single blind, the study was conducted as a double-blind, randomized, placebo-controlled study in two parts. Part 1 comprised of -ascending single oral doses and a food effect evaluation and Part 2 consisted of two different twice-daily oral doses (Table 1). Oral doses were chosen for both parts of the study, as this is the intended route of clinical administration.

**Table 1:** **Summary of dosing and group design for Parts 1 and 2 of the study**

**Part**	**Group (N)**	**SMT19969: Placebo**	**Dose and Interval**	**Dietary status**	**Subject numbers**
1	A (4)	3:1	2 mg Single	Fasted	101 to 104
	B (4)	3:1	20 mg Single	Fasted	105 to 108
	C (8)	6:2	100 mg Single	Fasted	109 to 116
	D (8)	6:2	400 mg Single	Fasted	117 to 124
	E TP1 (8)	6:2	1,000 mg Single	Fasted	125 to 132
	E TP2 (8)	6:2	1,000 mg Single	Fed	125 to 132
	F (8)	6:2	2,000 mg Single	Fasted	133 to 140
2	G (8)	6:2	200 mg BID	Fed	201 to 208
	H (8)	6:2	500 mg BID	Fed	209 to 216

*Abbreviations*: *BID* twice daily, *N* number of subjects studied, *TP* treatment period.

The primary objective was to determine the safety and tolerability of ascending single and multiple oral doses of SMT19969 in healthy male subjects. Safety and tolerability were assessed through adverse event monitoring and clinical laboratory evaluations as described below. The secondary objectives were to determine the single and multiple oral dose PK of SMT19969, to assess the effect of food on the systemic exposure of SMT19969, to assess the effect of multiple oral doses of SMT19969 on gut microbiota (via assessment of faecal samples) and to determine concentrations of SMT19969 in faecal samples. In addition, exploratory work was carried out on selected faecal samples to look for potential metabolites of SMT19969. Sampling time points and methodology for secondary objectives are described below.

### Part 1

Forty male subjects were studied in six groups (A to F) with each group receiving a single ascending oral dose of SMT19969 or placebo. Groups A and B consisted of four subjects (three receiving SMT19969 and one placebo), while each of Groups C to F had eight subjects with six receiving SMT19969 and two given placebo. Each subject in Groups A to D and Group F participated in one treatment period (TP) only whilst subjects in Group E (food effect) participated in two treatment periods. Subjects were required to be in residency from Day −1 (the day prior to first dose administration) and to remain for not less than 72 hours after each dosing occasion, apart from Group E TP2 in which subjects were required to remain resident until 24 hours post dose (Day 2). There was a minimum of at least 7 days between completion of dosing in one group and start of dosing in the next group to allow a satisfactory review of the safety and PK data from lower dose levels prior to progression to the next higher dose level [21].Six single oral dose levels of SMT19969 (2 to 2000 mg) or placebo were studied (Table 1).

In Part 1, the dose was administrated following an overnight fast on the morning of Day 1 except Group E TP 2, with dosing occurring 30 minutes after a high-fat breakfast [total energy content 895 Kcal; total fat content 61 g (61% of total calories); total protein 41 g (18% of total calories); total carbohydrate 46 g (19% of total calories)] on the morning of Day 1. Group E subjects received the same treatment (single oral dose of SMT19969 or placebo) in both TPs. All subjects in Part 1 returned for a post study visit 5 to 7 days after their final dose for the following safety assessments: Adverse event recording, blood pressure and pulse rate, oral body temperature, 12 lead ECG, clinical laboratory evaluations and physical examination.

### Part 2

Sixteen male subjects were randomized to two groups (G and H). In each group six subjects received SMT19969 and two were given placebo. Subjects were required to be in residency 2 days before first dose administration (Day −2) and to remain until Day 12 (48 hours post final dose). Two oral dose levels of 200 and 500 mg BID of SMT19969 or placebo were studied in Groups G and H respectively (Table 1). For Part 2, subjects received a twice daily (BID) oral dose of SMT19969 or placebo from Days 1 to 9 (12 hour interval), and a final single oral dose on the morning of Day 10. The morning doses were given 30 minutes after a light breakfast and the second daily doses were administered 50 minutes following an evening meal. All subjects in Part 2 returned for a post-study visit 5 to 7 days post final dose for the following safety assessments: Adverse event recording, blood pressure and pulse rate, oral body temperature, 12 lead ECG, clinical laboratory evaluations and physical examination.

### Study subjects

Inclusion criteria were that subjects were healthy males between 18 and 55 years of age with a body mass index (BMI) between 18.0 and 32 kg/m^2^. Subjects were excluded if they or their partners were unwilling to use appropriate contraception, or had received any prescribed systemic or topical medication within 14 days, or had used any non-prescribed systemic medication within 7 days (with the exception of paracetamol ≤2 g/day), or herbal supplements within 28 days. Subjects were also excluded if they had irregular bowel habits or had a positive faecal occult blood (FOB) at Screening and Day −1 (Groups C to F) or Screening and Day −2 (Part 2). Further exclusion criteria included cardiovascular disease, alcohol consumption >28 units per week, tobacco consumption >15 cigarettes per day or a clinically significant illness within 4 weeks of enrolment. All subjects underwent study-specific screening within 28 days prior to the first dose administration. Informed consent was obtained from all participants. Subject’s race was recorded as either American Indian or Alaska Native, Asian, Black, Native Hawaiian or Other Pacific Islander, White or Other. Where the race identify was recorded as Other, then additional specific information was noted.

### Randomisation and blinding

The treatment randomisation was produced by the Statistics Department at Covance CRU using a computer-generated pseudo-random permutation procedure. Two subjects were randomly assigned to receive placebo for Groups B-H whereas for Group A sentinel dosing was used. Subjects were dosed in numerical order according to the treatment randomisation. Subjects were enrolled by blinded clinical staff, whilst subjects were assigned to interventions by unblind pharmacy staff who prepared the unit doses for the subjects and kept a copy of the master treatment randomisation. Unit doses were given to the participants in a blinded manner by the clinical staff. The Investigator, clinical staff and data management staff remained blinded until database lock. To enable the Investigator to break the code, if required for safety reasons, individual sealed envelopes containing the treatment code for each subject were kept in the Covance CRU pharmacy. If it was necessary to break the code during the study, the date, time and reason would have been recorded in the subject’s source data and on the individual envelope.

### Pharmacokinetic assessments

#### Blood and faecal sampling

Approximately 2 mL of blood was collected at specific time points for each subject for quantification of SMT19969. In Part 1 blood was collected pre-dose and at 1, 2, 4, 8, 12 and 24 hours post-dose. In Part 2 blood was collected on Day 1 pre-am dose and at 2, 4, 8 and 12 hours post dose; Days 2 to 9 pre-am dose and Day 10 pre-dose and at 1, 2, 4, 8, 12, 24 and 48 hours post dose.

The following pharmacokinetic (PK) parameters were determined: area under the plasma concentration-time curve from time zero up to the last quantifiable concentration (AUC_0-tlast_), maximum observed plasma concentration (C_max_), time of maximum observed plasma concentration (t_max_). Pharmacokinetic parameters were determined using non-compartmental procedures and actual sampling times post dose used in the computation of PK parameters. Lower limit of quantification (LLOQ) was defined as 0.1 ng/mL. Due to the low levels and limited number of samples in which SMT19969 was detected, PK analysis conducted on ‘worst case’ scenario with plasma concentrations below the limit of quantification (BLQ) from the time of pre-dose sampling (t = 0) up to the time of the first quantifiable concentration set to a value of zero. The first BLQ value after a quantifiable level was replaced with the value of LLOQ (0.1 ng/mL). After this time point, BLQ plasma concentrations were set to zero. In Part 1 Groups C to F, faecal samples voided 0–72 hours post dose were pooled each 24-hour interval and analysed for concentration of SMT19969. During Part 2, all faecal samples voided whilst resident in the CRU were collected and stored in a freezer within 15 minutes of voiding pending analysis. Analysis of faecal and plasma samples was performed by CLE (Harrogate UK). Concentrations of SMT19969 in plasma samples were determined by liquid chromatography with tandem mass spectrometric detection (LC-MS/MS) following sample preparation by protein precipitation and on-line solid phase extraction. Faecal samples were homogenised (1:19 faeces: water/acetonitrile/methanesulfonic acid [60:40:1 v/v/v]) with concentrations of SMT19969 determined by LC-MS/MS. The faecal LLOQ was 20 μg/g.

Day 5 and Day 10 faecal samples from three subjects in Group G and three pre-dose samples from Group C (as a negative control) were analysed for the presence of metabolites using accurate mass LC-MS (Thermo LTQ Orbitrap mass spectrometer). Data were interrogated for the presence of metabolites based on accurate masses of potential metabolites using Metworks software (version 1.2) in conjunction with Xcalibur 2.0.7.

### Gut microbiota analysis

Pharmacodynamic measurements were performed on faecal samples in Part 2. Aliquots of the first faecal sample of the day voided on Days −1, 4 and 9 were stored at -70°C pending analysis of gut microbiota. All anaerobic culture and manipulations were performed in an Anaerobic Workstation (Don Whitley Scientific, Shipley, UK) at 37°C. A portion of each faecal sample (1 g) was diluted in 10 ml pre-reduced PBS to produce a 10% w/v faecal slurry.

For enumeration of *C. difficile* spores a 2 ml aliquot was removed which was treated with an equal volume of 96% ethanol and left for 1 h at room temperature. The alcohol shocked suspension was then 10-fold serially diluted to 10^−8^ in pre-reduced peptone water (Sigma Aldrich, UK). 20 μl of each dilution was used to inoculate CCEYL plates [Brazier’s CCEY agar, (Bioconnections, Leeds, UK) supplemented with 5 mg/L lysozyme and 2% lysed horse blood] in triplicate. Plates were incubated anaerobically for 48 h and single colonies were counted.

For enumeration of bacteroides, bifidiobacteria, lactobacilli, total clostridia, *C. difficile*, total anaerobes, lactose-fermenting enterobacteriaceae (LFE), enterococci and total aerobes an aliquot of each sample (500 μl) was serially diluted in 4.5 ml of pre-reduced peptone water to a dilution of 10^−9^ in an anaerobic cabinet. Selective agars as previously described [22] were inoculated in triplicate with 20 μl of each appropriate dilution. After incubation, colonies were counted and identified on the basis of colony morphology, colony fluorescence, Gram stain and biochemical reactivity. The limit of detection was 50 cfu/mL.

### Safety and tolerability assessments

#### Adverse events

Any adverse events or remedial actions were recorded in the subject’s electronic CRF (eCRF) and coded using Medical Dictionary for Regulatory Activities Version 16.0 terminology. The nature, time of onset, duration and severity were documented, together with the Investigator’s opinion of the relationship to drug administration.

### Vital signs

Supine blood pressure, supine pulse rate and oral body temperature were measured at specified times and also performed at other times if judged to be clinically appropriate.

### Electrocardiography

At specified specific times during the study and when judged to be clinically appropriate, a 12-lead resting electrocardiogram (ECG) with a 10-second rhythm strip was taken after the subject was supine for at least 5 minutes.

### Clinical laboratory evaluations

Blood and urine were collected for the following laboratory evaluations; serum biochemistry, haematology, serology (Hepatitis B surface antigen, Hepatitis C antibody, HIV), drug and alcohol screen and urinalysis.

### Faecal occult blood (FOB)

FOB (hema-screen, Alpha Labs, Eastleigh, UK) assessment was conducted on samples collected at screening and voided 48–72 hours post dose. Whilst in residence, all faecal samples were also visually assessed for consistency in accordance with the Bristol Stool Chart [23].

### Statistical analysis

The analysis population consisted of all subjects who received one or more doses of study drug. No formal statistical assessment of sample size was conducted, as this was the first time SMT19969 had been administered to man. However, the number of subjects who participated in this study is common in early clinical pharmacology studies and was considered sufficient to achieve the objectives of the study. No inferential statistical analyses were performed for PK.

The study was carried out in compliance with the Helsinki Declaration (http://www.wma.net/en/30publications/10policies/b3/index.html website). Ethical approval was received on 20/09/2012 (reference number 12/EE0362) from the National Research Ethics Service (NRES) Committee East of England.

## Results

**Figure 1:**
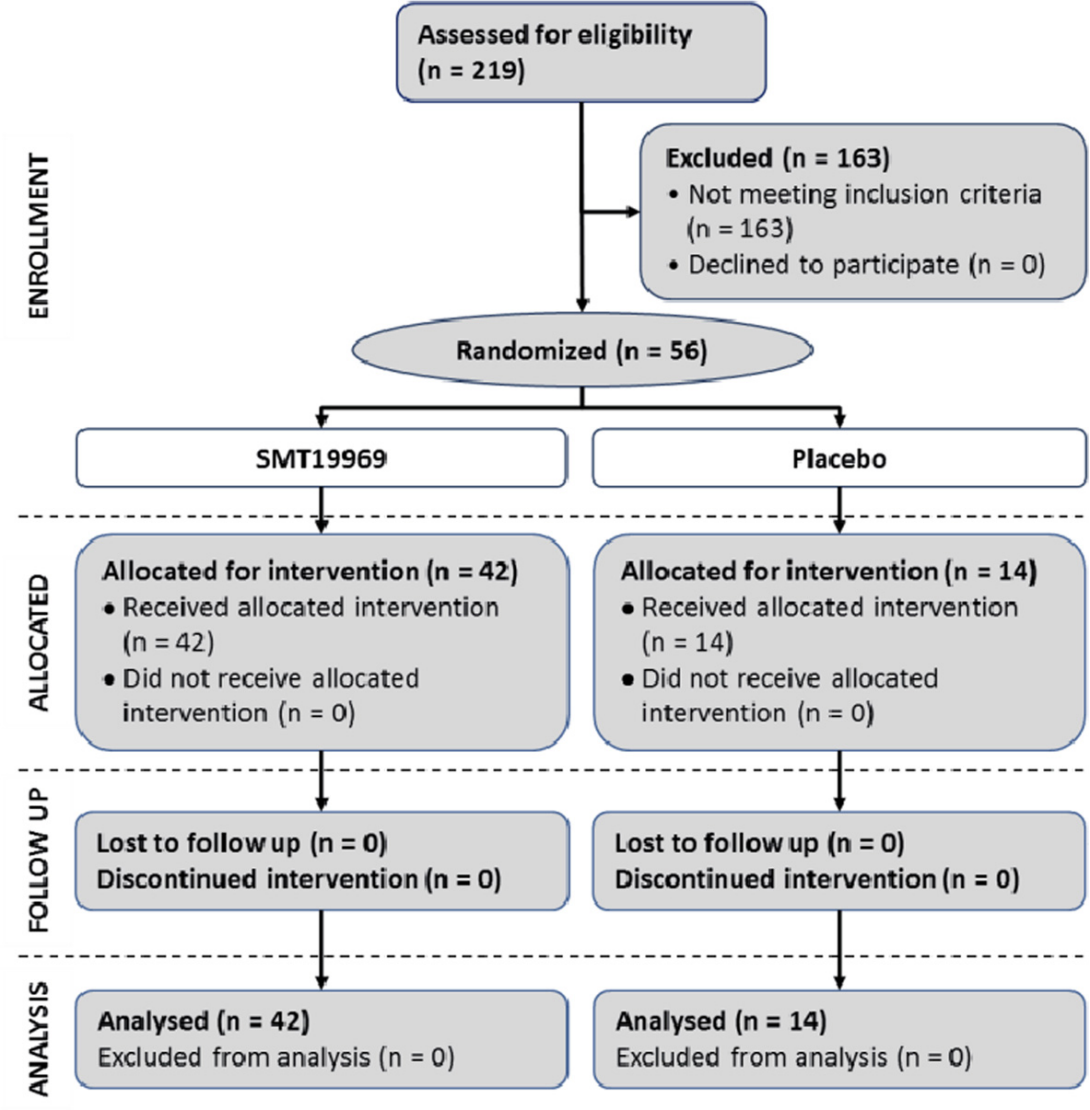
**Study flow diagram.**

A total of 56 subjects were randomized (Figure 1) and entered the study, with 40 subjects in Part 1, and 16 subjects in Part 2. The study started on 8th October 2012 (date of first informed consent) and was completed on 8th April 2013 once all subjects had been dosed and the final post study observation had been carried out. In Part 1, all subjects were men with a mean age of 32 years (range 18 to 53 years), a mean weight of 79.8 kg (range 54.7 to 99.2 kg), mean height of 178 cm (range 166 to 194 cm) and a mean BMI of 25.4 kg/m^2^ (range 19.4 and 31.3 kg/m^2^). All subjects were White, except one subject who was Black, two subjects who were Other: African/Caucasian, and one subject who was Other: mixed race - Caucasian and Asian. In Part 2, all subjects were men with a mean age of 35 years (range 18 to 54 years), a mean weight of 81.7 kg (range 62.8 to 96.0 kg), mean height of 179 cm (range 168 to 193 cm) and a mean BMI of 25.7 kg/m^2^ (range 18.3 to 30.3 kg/m^2^). All subjects were White, except one subject who was Asian. One subject in Group B was withdrawn from the study due to acute appendicitis, which commenced on Day 1 and was not considered due to study drug. A total of 55 subjects completed the study in accordance with the protocol and the treatment randomization. All 56 subjects were included in the safety population.

### Safety

Following single oral doses of SMT19969 in Part 1 and multiple oral doses of SMT19969 in Part 2, there were no apparent treatment- or dose-related trends in supine systolic and diastolic blood pressure, pulse rate and oral body temperature. There were no clinically important findings in the morphology of the 12-lead ECG for individual subjects at each single and multiple dose level of SMT19969. There were no clinically significant findings in the physical examination performed at Screening or at post-study. There were no treatment- or dose-related trends in the serum biochemistry, haematology, or urinalysis data during the study. All subjects in Part 1 Groups C to F and Part 2 tested negative for occult blood in post-dose faecal samples. The majority of faecal samples in both parts of the study were categorized as type 4 or below on the Bristol Stool Chart. Frequency and quality of stool was, in the opinion of the investigators, independent of dose.

Overall, all doses of SMT19969 were well tolerated when administered as single oral doses or BID oral doses for 10 days. The majority of AEs considered possibly related to study drug were classified as gastrointestinal (GI) disorders and were mild in severity and resolved without treatment (Table 2). Overall the incidence of adverse events reported by subjects in both Part 1 and Part 2 was low and no greater than the incidence of AEs reported by subjects administered placebo. There was one serious adverse event (SAE), acute appendicitis, which led to the subject being discontinued from the study and was considered unlikely to be related to the study drug.

**Table 2:** **Incidence of adverse events in Groups A to H**

		**Number of subjects with adverse events considered possible related to study drug**								
		**Group and dose of SMT19969**								
**Adverse event**	**A-H**	**A**	**B**	**C**	**D**	**E**		**F**	**G**	**H**
	**Placebo**	**2 mg**	**20 mg**	**100 mg**	**400 mg**	**1,000 mg**		**2,000 mg**	**200 mg BID**	**500 mg BID**
	**(N = 14)**	**(N = 3)**	**(N = 3)**	**(N = 6)**	**(N = 6)**	**TP1(N = 6)**	**TP2(N = 6)**	**(N = 6)**	**(N = 6)**	**(N = 6)**
Diarrhoea	3			1	2			1		2
Abdominal distension	1			1				1		
Abdominal pain	1							1		1
Duodeno-gastric reflux								1		
Flatulence	1				1					
Dyspepsia		1								
Feeling hot								1		
Paraesthesia									1	

*Abbreviations*: *BID* twice daily, *N* Number of subjects studied, *TP* Treatment period, *Blank cell* no AE reported.

### Pharmacokinetics in plasma

#### Part 1

Following single oral doses of up to 2,000 mg SMT19969 in the fasted state, plasma concentrations of SMT19969 were generally below the LLOQ of the assay (0.1 ng/mL) at the majority of blood sampling times. At the 100 and 2,000 mg dose levels, two and one subjects, respectively, had isolated plasma samples (one to two per subject) with quantifiable levels of SMT19969 that were close to the LLOQ of the assay (0.1 ng/mL) and ranged from 0.103 to 0.133 ng/mL. Following a single oral dose of 1,000 mg SMT19969 in the fed state, SMT19969 was quantified in the plasma in all six subjects (one to four quantifiable samples per subject), although levels were extremely low ranging from 0.102 to 0.296 ng/mL (Table 3). The time of maximum observed plasma concentration (T_max_) of SMT19969 occurred approximately 4 hours post-dose.

**Table 3:** **Summary of pharmacokinetic parameters following administration of SMT19969 in the fed state**

	**Mean (min** – **max) pharmacokinetic parameters**				
	**Group E TP2**	**Group G**		**Group H**	
	**1000 mg**	**200 mg BID**		**500 mg BID**	
	**Day 1**	**Day 1**	**Day 10**	**Day 1**	**Day 10**
	**(N = 6)**	**(N = 1)**	**(N = 5)**	**(N = 4)**	**(N = 6)**
AUC_0-tlast_ (ng.h/mL)	1.33 (0.706 – 3.46)	0.559 (N/A)	0.670 (0.524 – 1.30)	0.670 (0.531 – 1.23)	1.15 (0.515 – 1.98)
C_max_(ng/mL)	0.211 (0.102 – 0.296)	0.120 (N/A)	0.141 (0.108 – 0.243)	0.148 (0.110 – 0.305)	0.177 (0.105 – 0.279)

*Abbreviations*: *BID* twice daily, *AUC*_*0-tlast*_ area under the plasma concentration-time curve from time zero up to the last quantifiable concentration, *C*_*max*_ maximum observed plasma concentration, *N* Number of subjects from which SMT19969 concentrations were quantifiable and could be included in calculation of PK parameters.

#### Part 2

After repeat administration of SMT19969 in the fed state with 200 and 500 mg BID doses, parent compound was quantifiable in the plasma of most subjects by Day 10, although the plasma concentrations were very low, ranging from 0.105 to 0.305 ng/mL with T_max_ occurring at approximately 4 hours post-dose.

Pharmacokinetic parameters were only calculated from the groups dosed under fed conditions (Part 1 1,000 mg, Part 2 200 mg BID and 500 mg BID), as SMT19969 concentrations were quantifiable in one or more sample from the majority of subjects in these groups only. The exception was the 200 mg BID dose group on Day 1, in which only one subject had quantifiable concentrations of SMT19969 although these PK parameters were reported to enable comparison with Day 10 data. Table 3 summarizes the pharmacokinetic results for those subjects from which SMT19969 concentrations were quantifiable and could therefore be included in calculation of PK parameters.

Following repeat dosing the plasma concentration data suggest there may be some increase in absorption over time, with a greater proportion of subjects having quantifiable plasma levels of SMT19969 on Day 10, and the number of quantifiable samples per subject also being greater. This was suggested by a marginal increase in AUC_0-tlast_ on Day 10 compared to Day 1, although these data should be interpreted with care due to the non-standard, worst-case scenario approach used to derive these PK parameters. Overall, the systemic exposure of SMT19969 following oral dosing was minimal with plasma concentrations no more that approximately three-fold above the limit of quantification.

### Faecal sample analysis

Tables 4 and 5 present the results for faecal concentrations of SMT19969 determined during the two parts of the study. Following single and multiple oral doses of SMT19969, the mean daily faecal concentrations increased with increasing dose level. Following a single 400 mg dose, group mean faecal concentrations peaked at 1,132 μg/g 24–48 hours post dosing and remained high up to 72 hours post dosing (group mean = 677 μg/g). Multiple oral doses resulted in faecal concentrations significantly above the MIC_90_ of SMT19969 for *C. difficile* (0.125 – 0.25 μg/mL) with Day 10 Group G and H means (±SD) of 1,364 (407.07) and 3,318 (897.28) μg/mL, respectively.

**Table 4:** **Faecal concentrations of SMT19969 following single oral doses (Part 1)**

**Group**	**Dose**	**Mean (min** – **max) faecal concentrations (μg/g)**		
		**Time post dose (hours)**		
		**0-24**	**24-48**	**48-72**
C	100 mg	20 (<20 – 20)	213 (<20 – 330)	210 (<20 – 598)
D	400 mg	239 (<20 – 317)	1,132 (<20 – 3,340)	677 (436 – 948)
E TP1	1,000 mg	1,194 (<20 – 2,310)	855 (305 – 1,040)	1,209 (183 – 1,970)
F	2,000 mg	<20 (<20 – <20)	5713 (<20 – 11,800)	6,478 (1,030 – 17,600)

**Table 5:** **Faecal concentrations of SMT19969 following multiple oral doses (Part 2)**

**Group**	**Dose**	**Mean (min** – **max) Faecal concentrations (μg/g)**	
		**Nominal day of dosing**	
		**Day 5**	**Day 10**
G	200 mg twice daily	1,466 (847 – 2,390)	1,364 (783 – 1,980)
H	500 mg twice daily	2,084 (994 – 3,790)	3,318 (2,130 – 4,970)

No notable metabolites were detected in faeces, with the majority of the administered dose excreted as unchanged parent drug, which accounted for >97% of the total peak area. All metabolites detected were individually present at <0.3% of the total peak area. Table 6 presents the data for the composition of SMT19969 and metabolites from three subjects in Group G.

**Table 6:** **Composition of SMT19969 and metabolites in faeces of three subjects from Group G**

**Name**	**% of Total Peak Area**					
	**Subject 1**		**Subject 2**		**Subject 3**	
	**Day 5**	**Day 10**	**Day 5**	**Day 10**	**Day 5**	**Day 10**
SMT19969	99.05	99.34	99.21	100.00	97.99	97.72
Total metabolites	0.95	0.66	0.79	0.00	2.01	2.28

**Figure 2:**
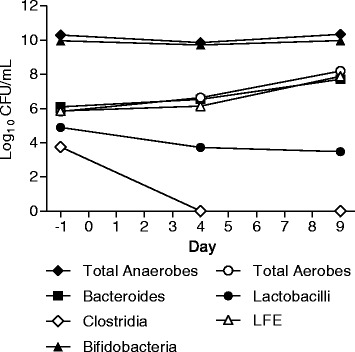
**Median log10 CFU/mL changes in gut microbiota composition for all Group G subjects.** Abbreviations: LFE = Lactose fermenting enterobacteriaceae.

**Figure 3:**
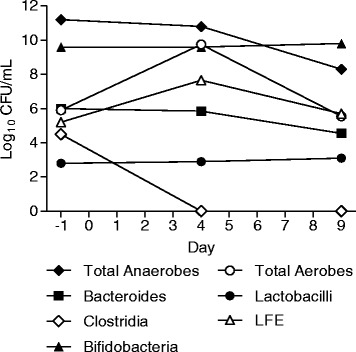
**Median log**_**10**_ **CFU/mL changes in gut microbiota composition for all Group H subjects.** Abbreviations: LFE = Lactose fermenting enterobacteriaceae.

Faecal samples voided pre and during the course of dosing in Group G and Group H were analysed for changes in gut microbiota composition. The panel of bacteria that were assessed were chosen as representative and reliably culturable Gram positive and Gram negative members of the endogenous gut microbiota and also to mimic the bacteria assessed in the human gut model of CDI [22,24]. Although culture techniques were used in this study, future studies examining the effect of SMT19969 on gut microbiota using genomic techniques are warranted. Overall the data show that repeat oral administration of SMT19969 caused minimal changes in bacterial counts (group median data shown in Figures 2 and 3), except for total clostridia where 3 log_10_ reductions in counts were observed in both Group G and H by day 4; clostridial counts remained below the limit of detection to day 9 (last day of measurement).

In Group G a slight increase was observed in bacteroides (0.5 log by day 4; 2 log by day 9) with a small decrease observed in the lactobacilli counts (1 log by day 4, which remained constant until day 9). Counts of bifidobacteria and total anaerobes remained constant with minimal fluctuations observed. Total aerobe and LFE counts both remained constant until day 4 and then both increased (2 log by day 9).

Comparable results were observed in Group H with SMT19969 having minimal effect on gut microbiota populations except for total clostridia. Total anaerobe and bacteroides populations remained constant until day 4, before decreasing modestly (2 log at day 9); bifidiobacteria and lactobacilli counts remained constant throughout. Numbers of total aerobes and LFEs increased (4 and 2 log, respectively) by day 4 before returning to baseline levels by day 9.

As part of the microbiota analysis, the quantification of enterococci was included although, particularly for Group G, counts were low or below the limit of detection. In Group H, enterococci were quantified in all pre-dose samples (median = 3.35 log_10_ CFU/mL), in half the day 4 samples (median = 1.1 log_10_ CFU/mL) and all day 9 samples except one (median = 2.7 log_10_ CFU/mL). In Group G, enterococci could only be quantified at all three time-points for one subject and at a single time-point (either pre-dose or day 4) for two other subjects. Conclusions on the potential effect of SMT19969 on enterococci are difficult to draw from these data although previous susceptibility testing [16] and data from a gut model of CDI [24] would indicate SMT19969 is associated with minimal activity against enterococci.

No *C. difficile* viable cells or spores were detected in any samples voided either pre-dosing or during the course of dosing.

## Discussion

CDI remains a significant burden to healthcare systems and new agents are required that effectively treat initial infection and reduce rates of recurrent disease. SMT19969 is a novel antimicrobial agent with a highly specific spectrum of activity that may cause reduced damage to the gut microbiota during CDI therapy compared with current standard of care. Such a restricted spectrum could be expected to maintain the majority of gut microbiota and so yield lower CDI recurrence rates.

This first in man study was designed to examine the safety, tolerability and pharmacokinetics of orally administered SMT19969. Overall, single oral doses of SMT19969 were considered to be safe and well tolerated by healthy male subjects when administered at doses levels of 2, 20, 100, 400, 1,000 and 2,000 mg. In addition, multiple oral doses of SMT19969 were considered to be safe and well tolerated by male subjects at dose levels of 200 and 500 mg BID for 9 days with a final dose on Day 10. All AEs considered possibly or likely due to study drug were mild and no dose dependent relationship between SMT19969 and incidence or severity of AEs was noted. No clinically significant findings from blood pressure, body temperature, 12-lead ECG, clinical laboratory evaluations (serum biochemistry, urinalysis and haematology), faecal occult blood or physical examination were observed.

Oral administration was associated with negligible systemic exposure. Although this increased following administration with food, the levels achieved were low and no more than approximately three fold above the limit of quantification with the highest recorded C_max_ = 0.305 ng/mL. The increase in systemic exposure of SMT19969 when administered with food is not likely to be of clinical significance. Following dosing at 200 mg BID, Day 5 and Day 10, achieved mean (± SD) faecal concentrations were 1,466 (±547) μg/g and 1,364 (±446) μg/g, respectively, which were significantly above the MIC for *C. difficile.*

The highly selective spectrum of activity of SMT19969 was confirmed by analysis of faecal samples obtained following repeat administration of SMT19969. With the exception of total clostridia, minimal disruption to the gut microbiota was observed. Notably, bacteroides, bifidobacteria and lactobacilli, which have been associated with colonisation resistance, were largely unaffected [10,25-27].These data indicate that SMT19969, unlike vancomycin and metronidazole, may not cause ongoing collateral damage to the gut microbiota during CDI therapy, allowing for natural restoration of colonisation resistance to be initiated during treatment. Such attributes may be expected to result in reduced rates of recurrent infection.

## Conclusions

In conclusion, this first in man study has shown oral administration of SMT19969 in healthy volunteers to be safe and well tolerated, to be associated with negligible oral bioavailability and to cause minimal disruption to gut microbiota. These results support continued clinical development of SMT19969 to further assess safety and efficacy as an oral therapy for CDI.
